# Sleep Regularity and Predictors of Sleep Efficiency and Sleep Duration in Elite Team Sport Athletes

**DOI:** 10.1186/s40798-022-00470-7

**Published:** 2022-06-17

**Authors:** Shona L. Halson, Rich D. Johnston, Laura Piromalli, Benita J. Lalor, Stuart Cormack, Gregory D. Roach, Charli Sargent

**Affiliations:** 1 School of Behavioural and Health Sciences, Australian Catholic University, McAuley at Banyo, Brisbane, Australia; 2 Sports Performance, Recovery, Injury and New Technologies (SPRINT) Research Centre, Australian Catholic University, Brisbane, QLD Australia; 3 Carnegie Applied Rugby Research Centre, Institute for Sport, Physical Activity and Leisure, Leeds Beckett University, Leeds, UK; 4 Western Australia Institute of Sport, Mt Claremont, Australia; 5 School of Behavioural and Health Sciences, Australian Catholic University, Melbourne, Australia; 6 Appleton Institute for Behavioural Science, Central Queensland University, Wayville, Australia

**Keywords:** Sleep regularity index, Athlete, Team sport, Variability

## Abstract

**Background:**

Many elite athletes have suboptimal sleep duration and efficiency, potentially due to factors that may impact sleep onset and offset times. Variability in sleep onset and offset may negatively influence sleep. The sleep regularity index (SRI) is a novel metric for sleep regularity, however there are no published descriptions of SRI in elite athletes. Further, contributors to sleep efficiency and duration in elite athletes using objective measures have not been explored.

**Methods:**

Sleep was monitored over a minimum of seven consecutive days (7 to 43)—in 203 elite team sport athletes (age range = 19–36 years; female, *n* = 79; male, *n* = 124, total sleep nights = 1975) using activity monitoring and sleep diaries. The sleep regularity index (SRI) was calculated to reflect the night-to-night shifts in sleep by accounting for changes in sleep onset and sleep offset. Sleep characteristics were compared between regular and irregular sleepers and important contributors to sleep efficiency and total sleep time were assessed using multiple linear regression models.

**Results:**

The median sleep regularity index and interquartile range were 85.1 (81.4 to 88.8). When compared to irregular sleepers, regular sleepers demonstrated (1) significantly greater sleep efficiency (*p* = 0.006; 0.31 *medium* effect size [ES]), (2) significantly less variability in total sleep time (− *p* ≤ 0.001; − 0.69, *large* ES) and sleep efficiency (− 0.34, *small* ES), (3) similar total sleep time and (4) significantly less variation in sleep onset (*p* ≤ 0.001; − 0.73, *large* ES) and offset (*p* ≤ 0.001; − 0.74, *large* ES) times. Sleep characteristics explained 73% and 22% of the variance in total sleep time and sleep efficiency, respectively. The most important contributor to total sleep time was a later sleep offset time, while the most important contributors to sleep efficiency were an earlier bedtime and less variable sleep onset times.

**Conclusions:**

Bedtime and a consistent sleep onset time are important factors associated with sleep efficiency in athletes, while sleep offset is an important factor for total sleep time. Coaches and staff can assist their athletes by providing training schedules that allow for both regularity and sufficiency of time in bed where possible.

## Key Points

- The median sleep regularity index and interquartile range in elite team sport athletes were 85 (80 to 88.3).
- SRI had a medium effect on sleep efficiency however, SRI had no impact on total sleep time.
- When compared to irregular sleepers, regular sleepers demonstrated significantly earlier sleep onset and offset times, and less variable total sleep time and sleep efficiency; there was no difference between groups in total sleep time.
- The most important contributor to total sleep time was a later sleep offset time, while the most important contributors to sleep efficiency were an earlier bedtime and less variable sleep onset times.
- Data from this study suggest that avoiding behaviours which may result in delayed sleep onset times may be important for sleep quality, while delaying sleep offset times may be important for sleep duration in elite athletes.

## Introduction

The importance of optimal sleep for athletes is becoming increasingly recognised; as such there is a concomitant increase in descriptive sleep data in athletes [1, 2]. On average, elite athletes have a lower than recommended sleep duration and sleep quality [1, 3–5], potentially due to training and competition times [6, 7], travel [8], stress [9], caffeine [10] and/or social media use [11]. Given the factors mentioned above, sleep onset and offset times in athletes may show substantial variation over time.

Most sleep research in athletes reports mean sleep characteristics over a specified monitoring period. However, calculation of sleep regularity or intraindividual variation is becoming popular in sleep science research [12, 13]. High variability in night-to-night sleep is related to objectively measured poor quality sleep, subjective sleep complaints and insomnia [14, 15]. Further, in a recent systematic review [16], daily intraindividual variability of sleep/wake patterns in the general population was associated with important physical and mental health outcomes. These included higher body mass index, weight gain, bipolar or depression symptomology, stress, symptoms of insomnia and poor sleep [16]. Investigating regularity of sleep, in elite athletes, may provide a more comprehensive understanding of athletes’ sleep behaviours. To date, only one study has examined sleep regularity in elite athletes, reporting that senior rugby league players exhibit less intraindividual variability in bedtime and sleep onset time compared with the junior level athletes. However, this study compared bed and wake time variation between senior and junior athletes and did not relate this variability to other aspects of sleep [17]. The sleep regularity index (SRI) has recently been proposed as a novel metric for sleep regularity [13]. The SRI calculates the percentage probability that an individual is in the same state (i.e. sleep vs. awake) at any two time points 24 h apart and to date has not been described in elite athletes. Given the potential variation in bed and wake times in elite athletes, and therefore sleep duration, the SRI appears an appropriate means to calculate and reflect the daily fluctuations in both sleep timing and sleep duration.

This is the first study of its kind to examine sleep regularity in a large cohort of male and female elite athletes and relate sleep regularity to sleep efficiency (indictor of sleep quality) and sleep duration. The aims of this study were to (1) report the SRI of 203 elite athletes over a minimum of 7 nights, (2) compare sleep characteristics between regular and irregular sleepers and (3) identify important contributors to sleep efficiency and duration, including regularity, in elite athletes. It is hypothesised that a greater daily variation in sleep duration, sleep onset and sleep offset times will result in reduced sleep efficiency in male and female elite athletes.

## Methods

### Participants

Sleep was monitored over a minimum of 7 nights in 203 athletes providing a total of 1975 nights of sleep. Professional elite athletes from four team sports (Netball, Australian Rules Football, Rugby League, Soccer) (age range = 19–36 years; female, *n* = 79; male, *n* = 124) gave informed consent to participate in the study. Participants were excluded if they were training or sleeping at altitude, if they were injured, if they reported a clinical diagnosis of a sleep disorder, or if they had undertaken transmeridian travel in the 2 weeks prior to data collection. All data collection occurred outside of competition periods and in the athlete’s typical training and sleep environment. Information on bed partners was not collected. The study was conducted in accordance with the standards of ethics outlined in the Declaration of Helsinki and was approved by the Australian Institute of Sport Human Research Ethics Committee.

### Procedures

Athletes’ sleep/wake behaviour was monitored using wrist activity monitors in conjunction with self-report paper sleep diaries. Each athlete wore an activity monitor on the same wrist throughout the data collection period, except when showering, swimming, or training. The sleep diaries were used to record two pieces of information for each night-time sleep: start date/time and end date/time. Daytime naps were not recorded. Athletes were instructed to complete their sleep diary each morning within 30 min after waking. There was no experimental manipulation of the athletes’ training schedules or sleep/wake behaviours and the athletes were free to consume nutritional supplements, caffeine or alcohol during the data collection period. Information regarding medication use (including sleeping pills) was not collected.

### Sleep Measurement

Two different models of activity monitor—produced by a sole manufacturer—were used in this study (Actiwatch-64 and Actical Z-series; Philips Respironics; Oregon, USA). Devices were configured to sum and store data in 1-min epochs based on activity counts from a piezoelectric accelerometer with a sensitivity of 0.05 g and a sampling rate of 32 Hz. Data from the sleep diary and activity monitor were used to determine when participants were awake and when they were asleep. Essentially, all time was scored as wake unless: (i) the sleep diary indicated that the athlete was lying down attempting to sleep and (ii) the activity counts from the monitor were sufficiently low to indicate that the athlete was immobile [26]. When these two conditions were satisfied simultaneously, time was scored as sleep. In this study, sensitivity was set at medium, which corresponds to a threshold activity count of 40. This scoring process was conducted using the Philips Respironics’ Actiwatch algorithm. Validation studies comparing wrist activity monitors with polysomnography report high levels of agreement in healthy adults (88%) [21] and well-trained athletes (81–90%). [22]

For each athlete, the following variables were derived for each sleep period:

- Bedtime (h:min): time at which the athlete attempted to initiate sleep;
- Sleep onset (h:min): the time at which an athlete first fell asleep after going to bed;
- Sleep offset (h:min): the time at which an athlete last woke before getting up;
- Sleep period (h): the time between sleep onset and sleep offset;
- Total sleep time: (h): the amount of sleep obtained during a sleep period;
- Sleep efficiency (%): total sleep time expressed as a percentage of the sleep period;
- Midpoint of sleep (h:min): time of the day at which the middle of the sleep period occurred;
- Sleep onset latency (min): the period of time between bedtime and sleep start.

### Data Analysis

All data and statistical analyses were conducted in RStudio (Version 1.1.463) using the R programming language (Version 4.0.5, *Shake and Throw*). Due to data being non-normally distributed, evidenced by significant Shapiro–Wilk tests (*p* < 0.05) and visual inspection of Q–Q plots, the median and interquartile range were used as measures of central tendency and dispersion, respectively.

The sleep regularity index (SRI) was calculated to reflect the night-to-night shifts in sleep cycles by accounting for changes in sleep onset and sleep offset over the longest interval of whole number of weeks (i.e. 7 days, 14 days, 21 days). The SRI is a metric that calculates the likelihood of sleep–wake cycles matching from 1 day to the next, which is then aggregated over a given period [13], using the following formula:

$${\text{SRI}} = - 100 + \frac{200}{{M\left( {N - 1} \right)}}\mathop \sum \limits_{j = 1}^{M} \mathop \sum \limits_{i = 1}^{N} \delta \left( {s_{i.j} , s_{i + 1.j} } \right)$$

where *N* is the number of days and *M* is the number of epochs per day (1-min epochs in this study). The function $\delta \left({s}_{i.j}, {s}_{i+1.j}\right)$ is equal to one, when the sleep–wake state is the same 24 h apart, otherwise zero. For example, if sleep occurred at 22:00 h on Monday and occurred at 22:00 h on Tuesday, then 1 was coded for the minute epoch. Only diurnal shifts in sleep onset and offset times were used to calculate SRI (i.e. daytime naps and wake after sleep onset were not assessed). Scores of 100 for SRI indicate that sleep–wake cycles are identical between days over the period, whereas a score of 0 would indicate no overlap between consecutive sleep–wake cycles. Participants were then classified as regular (*n* = 42) or irregular (*n* = 46) sleepers if they were in the top or bottom quintile based on their SRI score, respectively. Additionally, variability in sleep onset, offset, sleep efficiency and total sleep time were captured in two ways for each athlete; firstly, the median absolute deviation (MAD) was calculated to reflect individualised variation, relative to each individual’s median values. Secondly, absolute variation was calculated as the difference to the previous night for each variable. For example, if an individual had a sleep onset time of 22:00 h on night one and a sleep onset time of 21:30 h on night two, they would have a sleep onset variation of 30 min, these scores were then aggregated over the study period.

### Statistical Analyses

Aggregated data across all days were analysed for all participants. Differences in sleep characteristics between regular and irregular sleepers were assessed using the Wilcoxon rank sum test. The magnitude of differences were interpreted with effect sizes (*r*) and 95% confidence intervals as *trivial* ≤ 0.10 *small*, ≤ 0.3; *medium* ≤ 0.5; and *large*, > 0.5 [18] using the *rcompanion* package.

The influence of sleep behaviour over the data collection period on total sleep time and sleep efficiency was assessed using a number of machine learning algorithms to determine the most effective model. The algorithms included random forest regressions, elastic net regressions, boosted generalised additive models and multiple linear regressions using the *caret* package in R. Data were split into a training set (80%) and a testing set (20%). Subsequently, tenfold cross-validation with 5 repeats was used to train each model, with the final, tuned model being tested on the hold-out set. Separate models were built for total sleep time and sleep efficiency. Predictor variables included SRI, sleep onset time, sleep offset time, sleep midpoint time, individualised variation of sleep onset and offset (captured by the MAD), absolute sleep onset and offset variation: total sleep time and efficiency were also included in the efficiency and total sleep time models, respectively. The linear regression offered the best fit and accuracy on testing data, highlighted by the highest coefficient of determination (*R*^2^) and lowest normalised root mean square error (NRMSE; %), respectively. Variance inflation factor (VIF) was used to assess collinearity issues between variables, with a score of ≥ 10 used to remove variables; normality of the residuals was checked via a density plot.

## Results

**Fig. 1:**
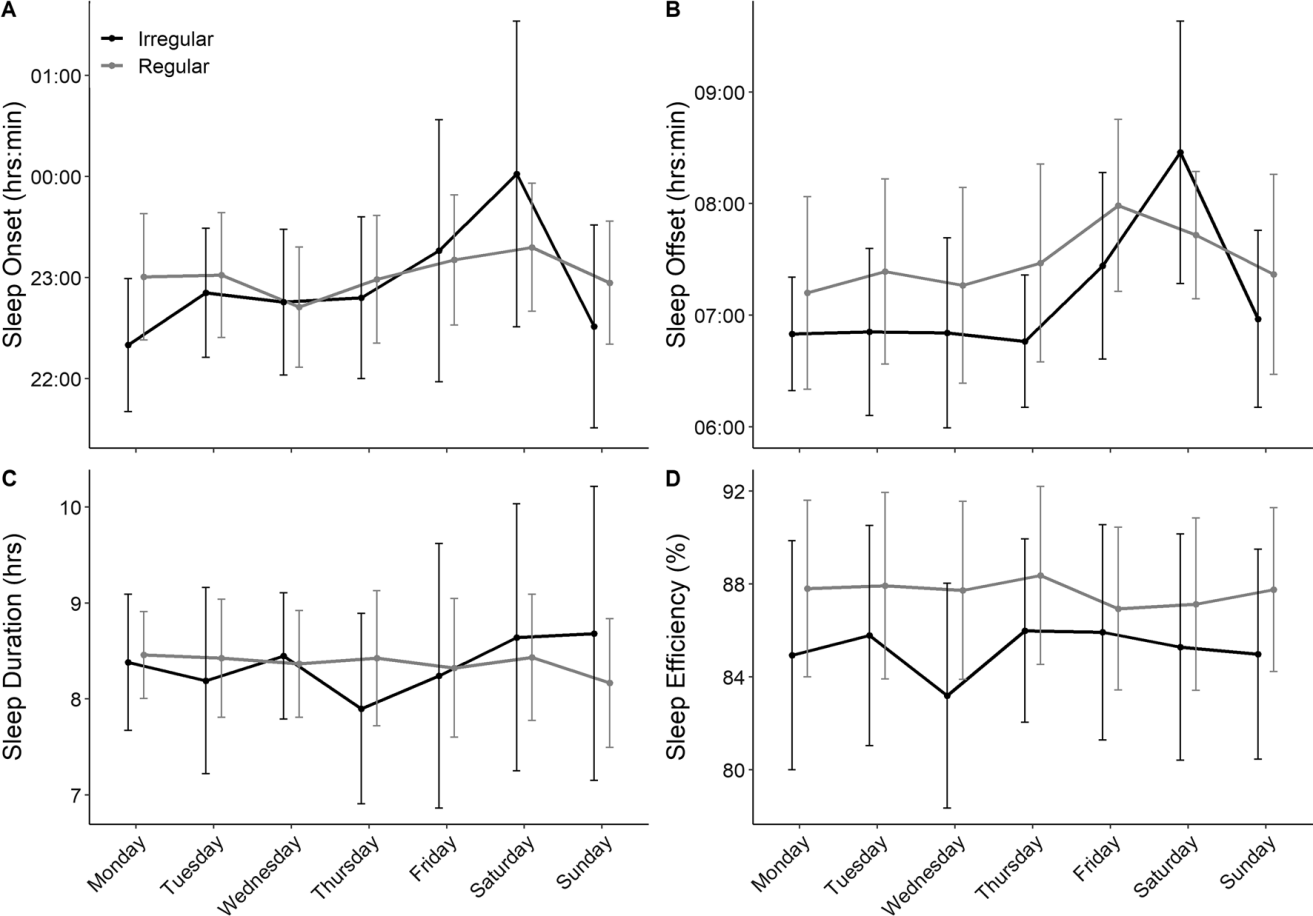
Daily sleep behaviours for **A** sleep onset time, **B** sleep offset time, **C** total sleep duration and **D** sleep efficiency for irregular and regular sleepers. Data are median and interquartile range

Table 1 shows the descriptive data for sleep characteristics across the entire group. There were no significant differences in sleep duration between regular and irregular sleepers, characterised by only *trivial* or *small* differences in bedtime, sleep onset, offset and midpoint. Further, there was no significant difference in average total sleep time, but there was less variation (*p* ≤ 0.001) in regular sleepers. Regular sleepers did have significantly better sleep efficiency and also less variation in sleep efficiency, with a *medium* effect size difference. There was no difference in sleep onset latency between groups, with only a *trivial* difference observed. The daily sleep characteristics for both regular and irregular sleepers is shown in Fig. 1. This figure highlights that there is little difference in sleep onset (Fig. 1A) other than later and more variable onset on Friday and Saturday in irregular sleepers. Further, there is a similar pattern observed for sleep offset (Fig. 1B). These similarities are reflected by similar sleep durations across each day of the week (Fig. 1C). Sleep efficiency is consistently higher in regular sleepers irrespective of week day (Fig. 1D).

**Table 1:** Sleep behaviour of the entire group and for regular and irregular sleepers

Variable	Entire group	Regular sleepers	Irregular sleepers	Regular vs. irregular sleepers	
				*p* value	Effect size (*r*)
Bedtime (h:min)	22:59 (22:17 to 23:45)	22:44 (22:23 to 23:41)	22:55 (22:00 to 23:44)	0.604	− 0.06 (− 0.26 to 0.15); *trivial*
Sleep onset (h:min)	23:08 (22:28 to 23:55)	22:54 (22:30 to 23:48)	23:03 (22:07 to 23:52)	0.701	− 0.04 (− 0.26 to 0.19); *trivial*
Sleep offset (h:min)	07:23 (06:42 to 08:13)	07:04 (06:47 to 08:30)	07:28 (06:22 to 08:05)	0.679	− 0.05 (− 0.25 to 0.17); *trivial*
Midpoint of sleep (h:min)	03:15 (02:39 to 03:59)	03:17(02:43 to 04:15)	03:00 (02:21 to 03:51)	0.805	0.03 (− 0.18 to 0.24); *trivial*
Sleep regularity index (AU)	85.1 (81.4 to 88.8)	90.1 (89 to 91.3)	76.5 (72.9 to 80)	< 0.001*	0.86 (0.84 to 0.86); *large*
Sleep onset variation (min^.^night^−1^)	44 (14 to 75)	27 (7 to 48)	75 (19 to 131)	< 0.001*	− 0.73 (− 0.81 to − 0.61); *large*
Sleep offset variation (min^.^night^−1^)	39 (9 to 70)	23 (6 to 40)	62 (12 to 112)	< 0.001*	− 0.74 (− 0.83 to − 0.62); *large*
Total sleep time (h:min)	8:17 (7:30 to 9:04)	8:18 (7:39 to 8:56)	8:22 (7:19 to 9:24)	0.506	− 0.07 (− 0.27 to 0.14); *trivial*
Total sleep time variation (min^.^night^−1^)	54 (15 to 93)	33 (8 to 58)	77 (17 to 137)	< 0.001*	− 0.69 (− 0.78 to − 0.51); *large*
Sleep efficiency (%)	86.5 (81.9 to 91)	87.7 (83.9 to 91.5)	85 (80.1 to 89.9)	0.006*	0.31 (0.12 to 0.48); *medium*
Sleep efficiency variation (%^.^night^−1^)	3.3 (1.1 to 5.5)	2.7(0.9 to 4.6)	4.2 (1.5 to 7)	0.001*	− 0.34 (− 0.52 to − 0.16); *medium*
Sleep onset latency (min)	5 (0 to 12.5)	4 (0 to 10.5)	4 (0 to 10.5)	0.746	0.04 (− 0.16 to 0.25); *trivial*

Data are presented as median (interquartile range); Irregular and regular sleepers were those in the bottom or top quintile for sleep regularity index, respectively

*Denotes a significant difference between regular and irregular sleepers; effect sizes (*r*) were interpreted as *trivial* ≤ 0.10 *small*, ≤ 0.3; *medium* ≤ 0.5; and *large*, > 0.5

### Total Sleep Time

**Fig. 2:**
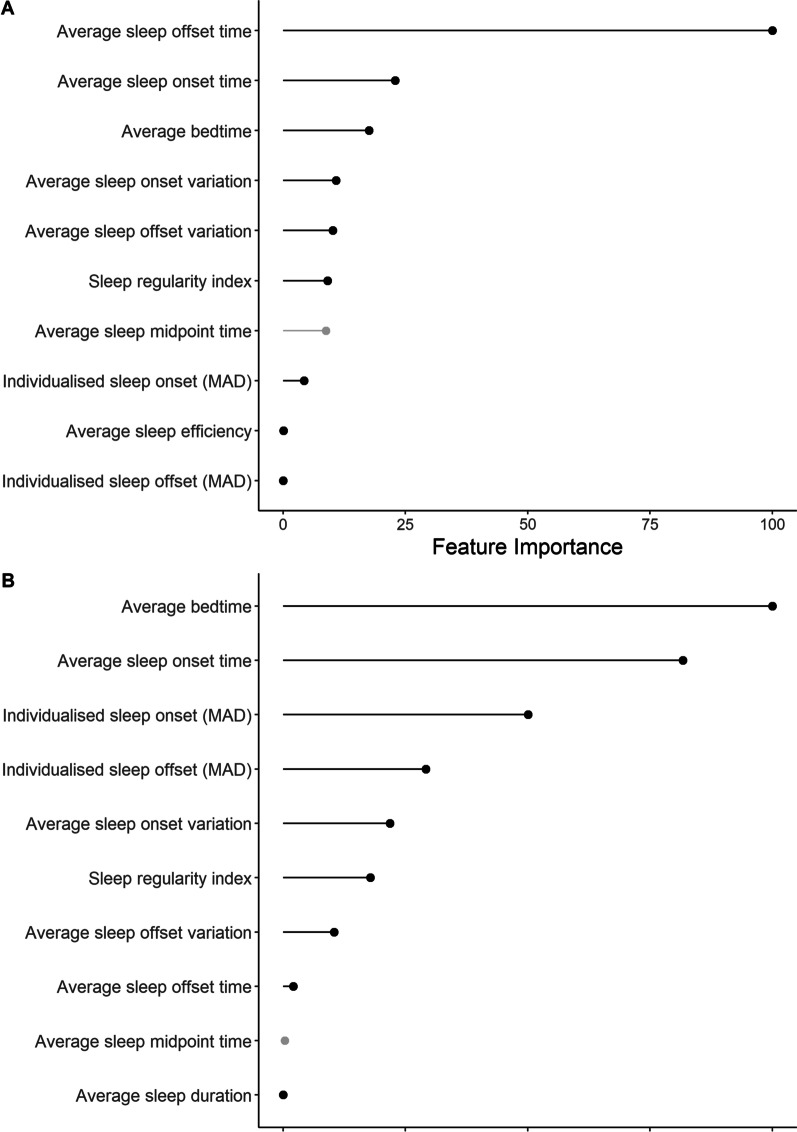
Importance of each variable for **A** average total sleep time and **B** average sleep efficiency over all days from the linear regression model. MAD = median absolute deviation; *Greyed out variables were removed from the final model due to collinearity issues (sleep midpoint both models, variance inflation factor [VIF] = 11.9)

**Fig. 3:**
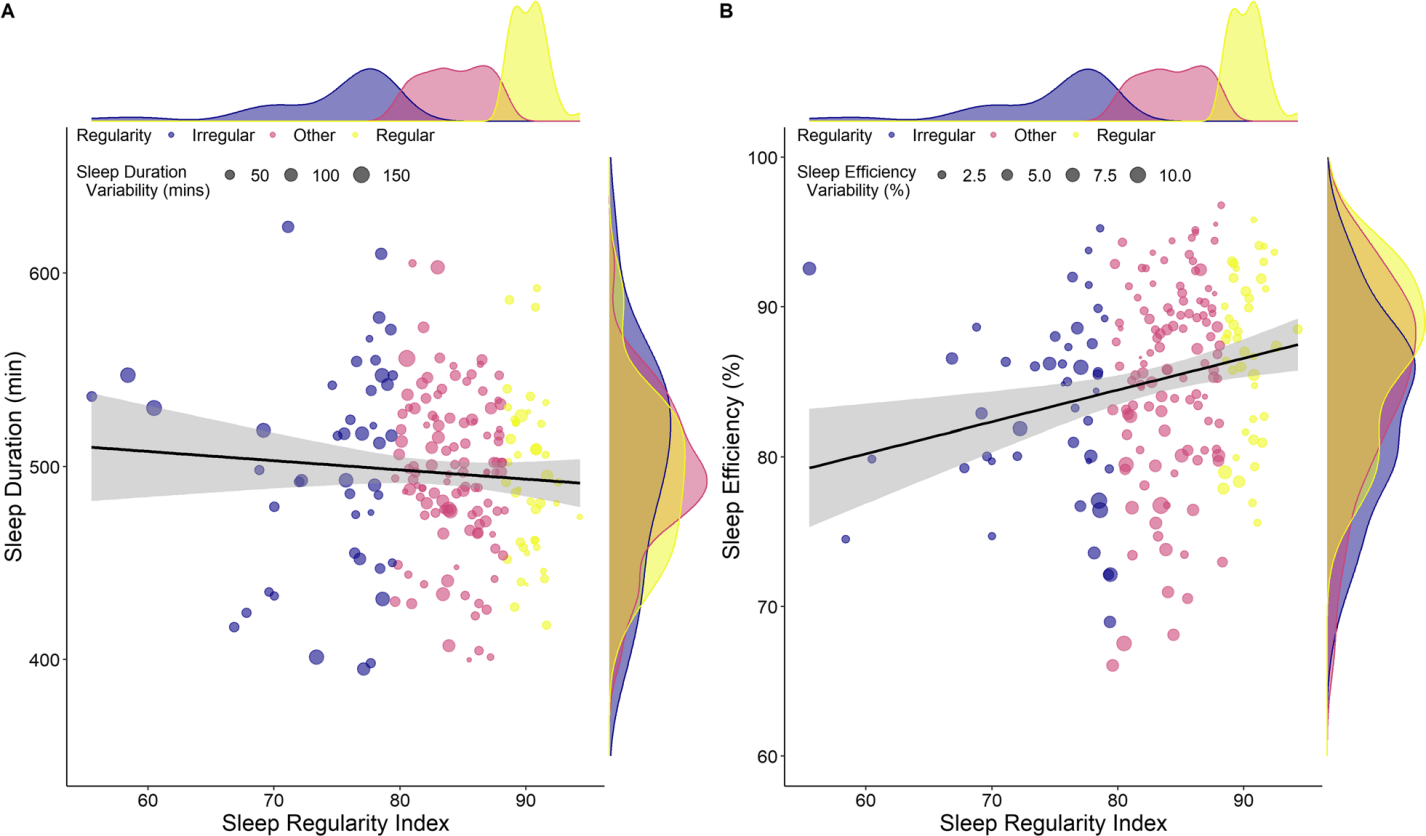
The relationship (and 95% confidence interval) between sleep regularity index and **A** sleep duration and sleep duration variation (MAD) and **B** average sleep efficiency and sleep efficiency variation (MAD); across all regular, irregular and other sleepers (a higher sleep regularity index score reflects more regular sleep cycles). The distribution of the data is also displayed on the plots

The linear regression for total sleep time achieved an *R*^2^ value of 0.79 on the training set and an RMSE of 21 min. On the test set, the model achieved an *R*^2^ of 0.78, explaining 78% of variance in total sleep time and a normalised RMSE of 3.6%. The most important predictor was sleep offset time, followed by sleep onset and bedtime. The remaining variables had little effect on total sleep time; sleep regularity had no effect on model accuracy (Fig. 2A). The relationship between SRI and total sleep time in Fig. 3A highlights the lack of any association between sleep regularity and total sleep time.

### Sleep Efficiency

**Fig. 4:**
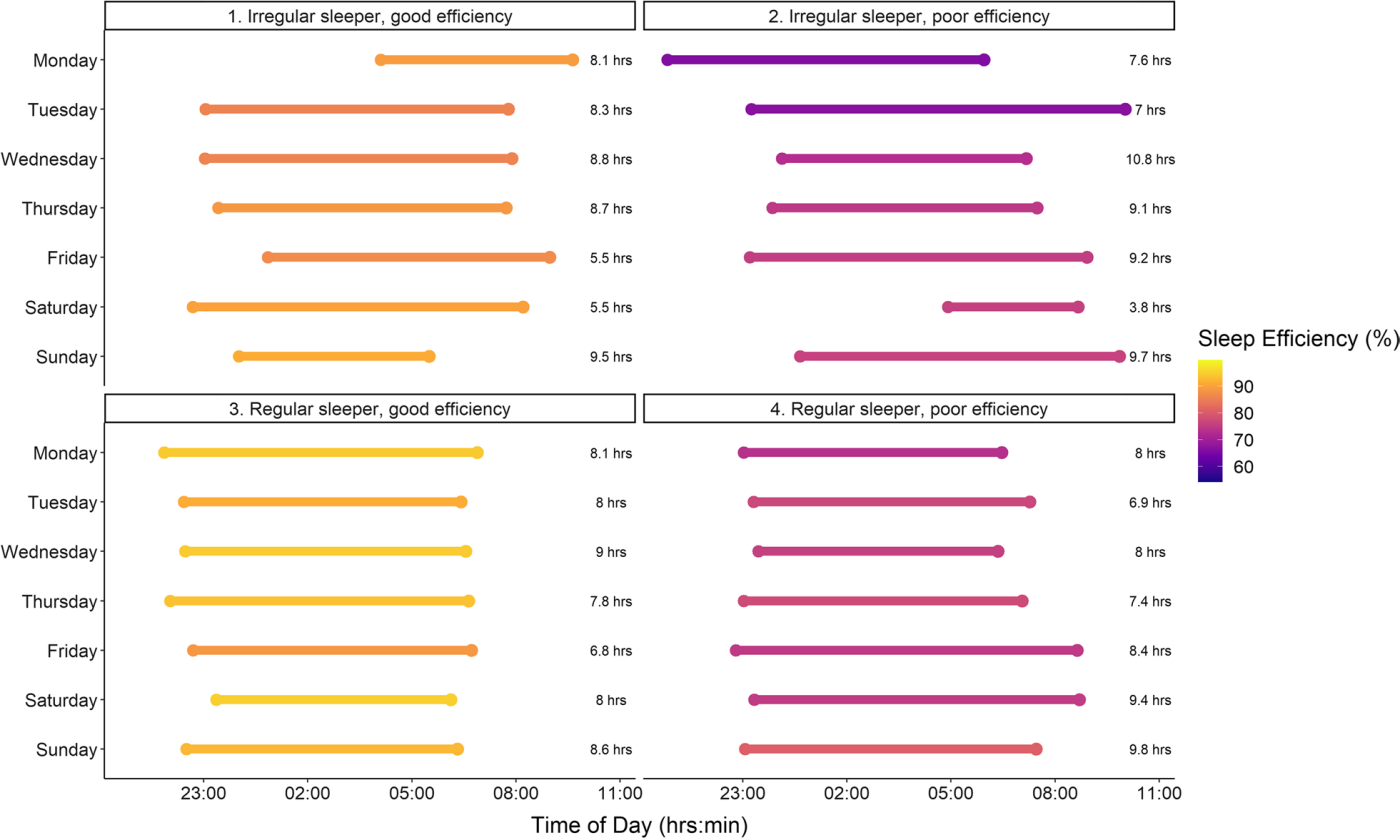
Individual sleep profiles for four athletes, that selected to reflect the individual nature of the relationships showing a (1) irregular sleeper with good sleep efficiency; (2) irregular sleeper with poor sleep efficiency; (3) regular sleeper with good sleep efficiency; (4) regular sleeper with poor sleep efficiency. Each bar reflects total sleep time with the colour highlighting sleep efficiency across each day of the week

On the training set, the linear regression achieved an *R*^2^ value of 0.15 and RMSE of 6.5%. On the hold-out test set, the final model achieved an *R*^2^ of 0.22, explaining 22% of variance in sleep efficiency with a normalised RMSE of 5.8%, showing moderate predictive performance of predictors on sleep efficiency. The importance of the predictors is shown in Fig. 2B; the most important predictors to sleep efficiency were average bedtime, sleep onset time, followed by individualised sleep onset and offset (MAD), average sleep onset variation and SRI. The relationship between SRI and sleep efficiency is shown in Fig. 3B with four profiles used to highlight the individual nature of sleep regularity and efficiency, where regular sleepers can have poor sleep efficiency and vice versa as shown in Fig. 4.

## Discussion

The aims of this study were to (i) assess sleep regularity over a minimum of 7 nights in elite athletes, (ii) compare sleep characteristics between regular and irregular sleepers and (iii) identify important contributors to total sleep time and sleep efficiency in elite athletes. The main findings of the study are that (a) the athletes in this study were good sleepers, with an average sleep duration of more than 8 h and an average sleep efficiency of 86.5%; (b) the median sleep regularity index was 85.1 (81.4 to 88.8), which is similar to data reported in both Australian University students (SRI: 79.85 ± 8.6) [19] and US college students (SRI: 73 ± 11) [13]; and (c) the SRI had no impact on total sleep time, but did influence sleep efficiency. When compared to irregular sleepers, regular sleepers demonstrated, (1) significantly greater sleep efficiency, (2) significantly less variability in total sleep time and sleep efficiency, (3) similar total sleep time and (4) significantly less variation in sleep onset (*p* ≤ 0.001; − 0.73, *large* ES) and offset (*p* ≤ 0.001; − 0.74, *large* ES) times. Sleep characteristics explained 73% and 22% of the variance in total sleep time and sleep efficiency, respectively. The most important contributor to total sleep time was a later sleep offset time, while the most important contributors to sleep efficiency were an earlier bedtime and less variable sleep onset times. Bedtime and a consistent sleep onset time are important factors associated with sleep efficiency in athletes, while sleep offset is an important factor for total sleep time. Coaches and staff can assist their athletes by providing training schedules that allow for both regularity and sufficiency of time in bed where possible.

Due to the relatively recent development of the SRI, there are no studies that have reported this metric in athletes and additionally, there are few studies that have reported this metric in populations of a similar age to the current study. Phillips et al. [13] reported a mean SRI in Australian university students similar to the median SRI of athletes in the present study and reported that sleep regularity was independent of sleep duration, with no difference in sleep duration when comparing regular to irregular sleepers [13]. A consistent weekend sleep offset time has been reported to result in a longer weekday sleep duration in non-shift working adults (age 23–48 years) using questionnaires and self-report [20]. Many elite athletes will have at least one non-training day per week (typically Sunday), thereby increasing the likelihood that they will have later sleep offset times on this day. This has the potential to increase the SRI in this population.

Despite the difference in sleep efficiency between regular and irregular sleepers, SRI had little predictive effect on sleep efficiency, in fact, sleep behaviours accounted for 22% of the variance in sleep efficiency. Further, sleep efficiency was largely homogenous in the group, with an IQR ranging from 82 to 91%, making predictive modelling difficult due to a lack of variance in the data. In spite of this, (early) bedtime was the most important factor influencing sleep efficiency. This was followed by individualised sleep onset (MAD), average sleep onset variation, average sleep onset time and average sleep duration. As such, while sleep behaviours do not explain all the variance in sleep efficiency, it does highlight that bedtime and low variability in sleep onset times may be important for sleep quality in athletes. Indeed, as shown in Fig. 1, regular sleepers showed relatively consistent onset and offset times, irrespective of the day of the week, in comparison with irregular sleepers. It should be noted that the major variation in sleep onset and offset times occurred on Friday and Saturday which may be due in part to a rest day on Sunday and/or behavioural factors that may be within the control of the athlete. However, it is not always possible for athletes to initiate sleep at a prescribed or specific time, which may result in large variability in sleep onset times, as shown by Athletes one and two in Fig. 3. This may be due to late training or competition times, other evening commitments, or they may not be biologically able to sleep at a certain time. Encouraging earlier bedtimes may also result in sleep onset issues if athletes are trying to ‘force’ sleep. Education regarding minimising behaviours that may contribute to inappropriate sleep onset times, may improve sleep quality in elite athletes. The most important factor identified for total sleep time was a later sleep offset time. Less important factors included: bedtime, sleep onset time and sleep efficiency. The finding of the importance of later sleep offset times is supported by research suggesting that early morning training sessions are associated with reduced sleep durations in elite athletes [21]. Further, athletes may have significantly later sleep offset times on non-training days, seemingly to ‘catch-up’ on sleep lost on training days [21]. Delaying training start times should increase sleep opportunity and also potentially reduce the discrepancy between training and non-training days. It is acknowledged that access to facilities, timing of travel and competition may influence the ability to optimally schedule sleep opportunities for athletes. However, delayed training start times, when possible, may be a potential means of protecting sleep in elite athletes.

The biological bases of sleep, driven by the two-process model of sleep (homeostatic drive and the circadian clock) are relatively stable across days in individuals following a diurnal sleep/wake schedule [16]. However, sleep regularity can be affected by other factors, many of which may be identified and potentially modified. Factors such as scheduling of training, psychological stress, stimulant use (caffeine) and societal and cultural influences may impact on sleep regularity and provide insight into the aetiology of poor sleep in athletes. Data from this study suggest that avoiding behaviours which may result in delayed sleep onset times may be important for sleep quality, while delaying sleep offset times may be important for sleep duration in elite athletes.

## Limitations

It should be noted that the SRI used in the present study reflects the shifts in night-time sleep onset and sleep offset and does not consider daytime naps or wake bouts throughout the night. Future work should establish the influence of all sleep episodes on SRI. Athletes in the current study were not competing, therefore removing an important potential contributor to variability in sleep onset and offset times [22–25]. It is likely that due to evening competition and therefore late bedtimes for many elite team sports, the SRI may be higher during competition periods. Other information such as bed partners and sleep medication use were not collected. Further, other periods of potential disruption such as transmeridian travel did not occur during the assessment period. Including these factors would have likely increased group heterogeneity and may have improved predictive performance of sleep behaviours on sleep. While sleep regularity had no effect on total sleep time, it is possible that sleep regularity may affect other factors relating to athletes' performance. This may include pre-training fatigue, daytime sleepiness and/or mental fatigue, which were not measured in this study. Finally, examining the association between sleep regularity and outcome measures such as illness, injury and performance will aid in determining relative importance of sleep regularity in elite athletes.

## Conclusion

In summary, regular sleepers had greater sleep efficiency, less variability in sleep efficiency and total sleep time, but similar total sleep time compared with irregular sleepers. Bedtime, sleep onset and sleep offset times influence sleep efficiency and total sleep time in athletes. While sleep behaviours only explained 22% of the variance in sleep efficiency, at the elite level, small changes may have large consequences for performance outcomes. As such, coaches and staff can assist their athletes by providing training schedules that allow for both regularity and sufficiency of time in bed where possible.

## Data Availability

The authors are willing to discuss data sharing under collaborative agreements. Please contact the corresponding author.

## References

[1] Walsh NP, Halson SL, Sargent C (2020). Sleep and the athlete: narrative review and 2021 expert consensus recommendations. Br J Sports Med.

[2] Sargent C, Lastella M, Halson SL, Roach GD (2021). How much sleep does an elite athlete need?. Int J Sports Physiol Perform.

[3] Leeder J, Glaister M, Pizzoferro K, Dawson J, Pedlar C (2012). Sleep duration and quality in elite athletes measured using wristwatch actigraphy. J Sports Sci.

[4] Gupta L, Morgan K, Gilchrist S (2017). Does elite sport degrade sleep quality? A systematic review. Sports Med.

[5] Halson SL, Johnston RD, Appaneal RN (2021). Sleep quality in elite athletes: normative values, reliability and understanding contributors to poor sleep. Sports Med.

[6] Lalor BJ, Halson SL, Tran J, Kemp JG, Cormack SJ (2020). A complex relationship: sleep, external training load, and well-being in elite Australian footballers. Int J Sports Physiol Perform.

[7] Lastella M, Roach GD, Vincent GE, Scanlan AT, Halson SL, Sargent C (2020). The impact of training load on sleep during a 14-day training camp in elite, adolescent, female basketball players. Int J Sports Physiol Perform.

[8] Janse van Rensburg DC, Jansen van Rensburg A, Fowler PM (2021). Managing travel fatigue and jet lag in athletes: a review and consensus statement. Sports Med.

[9] Halson SL, Appaneal RN, Welvaert M, Maniar N, Drew MK (2021). Stressed and not sleeping: poor sleep and psychological stress in elite athletes prior to the Rio 2016 Olympic games. Int J Sports Physiol Perform.

[10] Caia J, Halson SL, Holmberg PM, Kelly VG (2021). Does caffeine consumption influence postcompetition sleep in professional rugby league athletes? A case study. Int J Sports Physiol Perform.

[11] Jones MJ, Dawson B, Gucciardi DF (2019). Evening electronic device use and sleep patterns in athletes. J Sports Sci.

[12] Shoji KD, Tighe CA, Dautovich ND, McCrae CS (2015). Beyond mean values: quantifying intraindividual variability in pre-sleep arousal and sleep in younger and older community-dwelling adults. Sleep Sci.

[13] Phillips AJK, Clerx WM, O’Brien CS (2017). Irregular sleep/wake patterns are associated with poorer academic performance and delayed circadian and sleep/wake timing. Sci Rep.

[14] Lemola S, Ledermann T, Friedman EM (2013). Variability of sleep duration is related to subjective sleep quality and subjective well-being: an actigraphy study. PLoS ONE.

[15] Molzof HE, Emert SE, Tutek J (2018). Intraindividual sleep variability and its association with insomnia identity and poor sleep. Sleep Med.

[16] Bei B, Wiley JF, Trinder J, Manber R (2016). Beyond the mean: a systematic review on the correlates of daily intraindividual variability of sleep/wake patterns. Sleep Med Rev.

[17] Caia J, Halson SL, Scott TJ, Kelly VG (2017). Intra-individual variability in the sleep of senior and junior rugby league athletes during the competitive season. Chronobiol Int.

[18] Cohen J (1988). Statistical power analysis for the behavioural sciences.

[19] Windred DP, Stone JE, McGlashan E, Cain SW, Phillips A (2021). Attitudes towards sleep as a time commitment are associated with sleep regularity. Behav Sleep Med.

[20] Soehner AM, Kennedy KS, Monk TH (2011). Circadian preference and sleep-wake regularity: associations with self-report sleep parameters in daytime-working adults. Chronobiol Int.

[21] Sargent C, Halson S, Roach GD (2014). Sleep or swim? Early-morning training severely restricts the amount of sleep obtained by elite swimmers. Eur J Sport Sci.

[22] Fullagar HH, Skorski S, Duffield R, Julian R, Bartlett J, Meyer T (2016). Impaired sleep and recovery after night matches in elite football players. J Sports Sci.

[23] Sargent C, Roach GD (2016). Sleep duration is reduced in elite athletes following night-time competition. Chronobiol Int.

[24] O'Donnell S, Bird S, Jacobson G, Driller M (2018). Sleep and stress hormone responses to training and competition in elite female athletes. Eur J Sport Sci.

[25] Juliff LE, Peiffer JJ, Halson SL (2018). Night games and sleep: physiological, neuroendocrine, and psychometric mechanisms. Int J Sports Physiol Perform.

[26] Roach GD, Schmidt WF, Aughey RJ, Bourdon PC, Soria R, Claros JCJ, Garvican-Lewis LA, Buchheit M, Simpson BM, Hammond K, Kley M, Wachsmuth N, Gore CJ, Sargent C (2013). The sleep of elite athletes at sea level and high altitude: a comparison of sea-level natives and high-altitude natives (ISA3600). Br J Sports Med..

